# Supplementary material for: Polyethylene Terephthalate Microplastics Generated from Disposable Water Bottles Induce Interferon Signaling Pathways in Mouse Lung Epithelial Cells
Source: Nanomaterials (Basel). 2024 Jul 31;14(15):1287. doi: 10.3390/nano14151287 (PMC11314056; doi:10.3390/nano14151287)
Supplement: Supplementary file 1 [file nanomaterials-14-01287-s001.zip › Supplementary Tables and Figures.pdf]

Article

Polyethylene Terephthalate Microplastics Generated from Disposable Water Bottles Induce Interferon Signaling Pathways in Mouse Lung Epithelial Cells

Luna Rahman [1], Andrew Williams [1], Dongmei Wu [1] and Sabina Halappanavar [1,2,*]

[1] Environmental Health Science and Research Bureau, Health Canada, Ottawa, ON K1A 0K9, Canada; luna.rahman@hc-sc.gc.ca (L.R.); andrew.williams@hc-sc.gc.ca (A.W.); dongmei.wu@hc-sc.gc.ca (D.W.)

[2] Department of Biology, University of Ottawa, Ottawa, ON K1N 6N5, Canada

* Correspondence: sabina.halappanavar@hc-sc.gc.ca

Supplementary information

Table S1: Conditions for sample preparation for DLS analysis

MPs	Solvent for stock preparation	Solvent for dilution	Sample preparation	Final Concentration
**60nm PS**	MilliQ water	MilliQ water	5 min water bath sonication	50 μg.ml^{-1}
	MilliQ water	Cell culture media	5 min water bath sonication	50 μg.ml^{-1}
**100nm PS**	MilliQ water	MilliQ water	5 min water bath sonication	50 μg.ml^{-1}
	MilliQ water	Cell culture media	5 min water bath sonication	50 μg.ml^{-1}
**300nm PS**	MilliQ water	MilliQ water	5 min water bath sonication	50 μg.ml^{-1}
	MilliQ water	Cell culture media	5 min water bath sonication	50 μg.ml^{-1}
**PMMA**	MilliQ water	MilliQ water	20 min water bath sonication	100 μg.ml^{-1}
	MilliQ water	Cell culture media	20 min water bath sonication	1 mg.ml^{-1}
**LDPE**	MilliQ water	MilliQ water	Soaked in water for 2 h, then 40 min water bath sonication	1 mg.ml^{-1}
	Cell culture media	Cell culture media	Soaked in DMEM for 2 h, then 40 min water bath sonication	1 mg.ml^{-1}
**HDPE**	MilliQ water	MilliQ water	10 min water bath sonication	1 mg.ml^{-1}
	Cell culture media	Cell culture media	10 min water bath sonication	1 mg.ml^{-1}
**Nylon from teabag**	MilliQ water	MilliQ water	20 min water bath sonication	250 μg.ml^{-1}
	MilliQ water	Cell culture media	20 min water bath sonication	250 μg.ml^{-1}
**Nylon ≤2.7 μm fractionated from powder**	MilliQ water	MilliQ water	20 min water bath sonication	1 mg.ml^{-1}
	MilliQ water	Cell culture media	20 min water bath sonication	1 mg.ml^{-1}
**PETE Small**	MilliQ water	MilliQ water	20 min water bath sonication	1 mg.ml^{-1}
	MilliQ water	Cell culture media	20 min water bath sonication	1 mg.ml^{-1}
**PETE Large**	MilliQ water	MilliQ water	20 min water bath sonication	1 mg.ml^{-1}
	MilliQ water	Cell culture media	20 min water bath sonication	1 mg.ml^{-1}
**PP**	MilliQ water	MilliQ water	30 min water bath sonication of sample diluted from 10 mg.ml^{-1} stock in H_2O prepared with 60 min water bath sonication	1 mg.ml^{-1}
	Cell culture media	Cell culture media	30 min water bath sonication of sample diluted from 10 mg.ml^{-1} stock in cell culture media prepared with 60 min water bath sonication	1 mg.ml^{-1}

Table S2: Endotoxin level in-lab generated MPs.

Sample (No Spin Down)	Endotoxin (EU.mL^{-1})	Endotoxin SD	0.5 EU.mL^{-1} Spike % Recovery
**Nylon≤2.7 μm, teabag**	0.008482	0.002179	
**PETE≤2.7 μm**	0.005215	8.72E-05	
**PMMA**	0.004352	8.72E-05	
**Nylon + 0.5 EU Spike**	0.068458	0.008194	119.9532
**PETE + 0.5 EU Spike**	0.055329	0.001569	100.2281
**PMMA + 0.5 EU Spike**	0.053233	0.004882	97.76244

Maximum sensitivity of the measurement was 0.01 EU.mL^{-1}

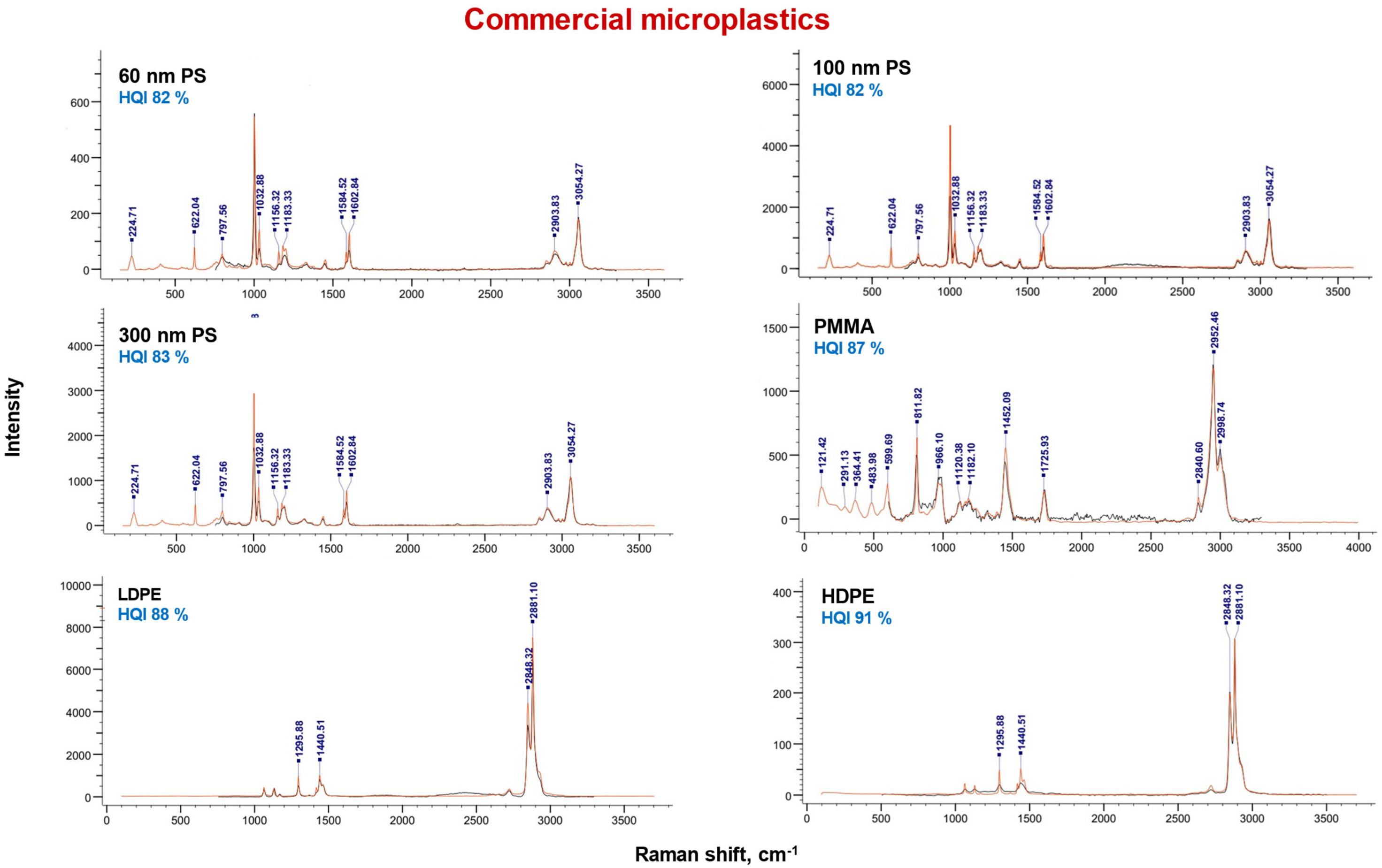

Figure S1. Raman spectra of commercial MPs. The black line represents the spectrum of the sample collected by Labspec6 and the orange line represents the spectrum of the known polymer from the database to which the sample spectrum matched efficiently. The hit quality index (HQI) describes the correlation between the sample spectrum and spectrum of the known polymer.

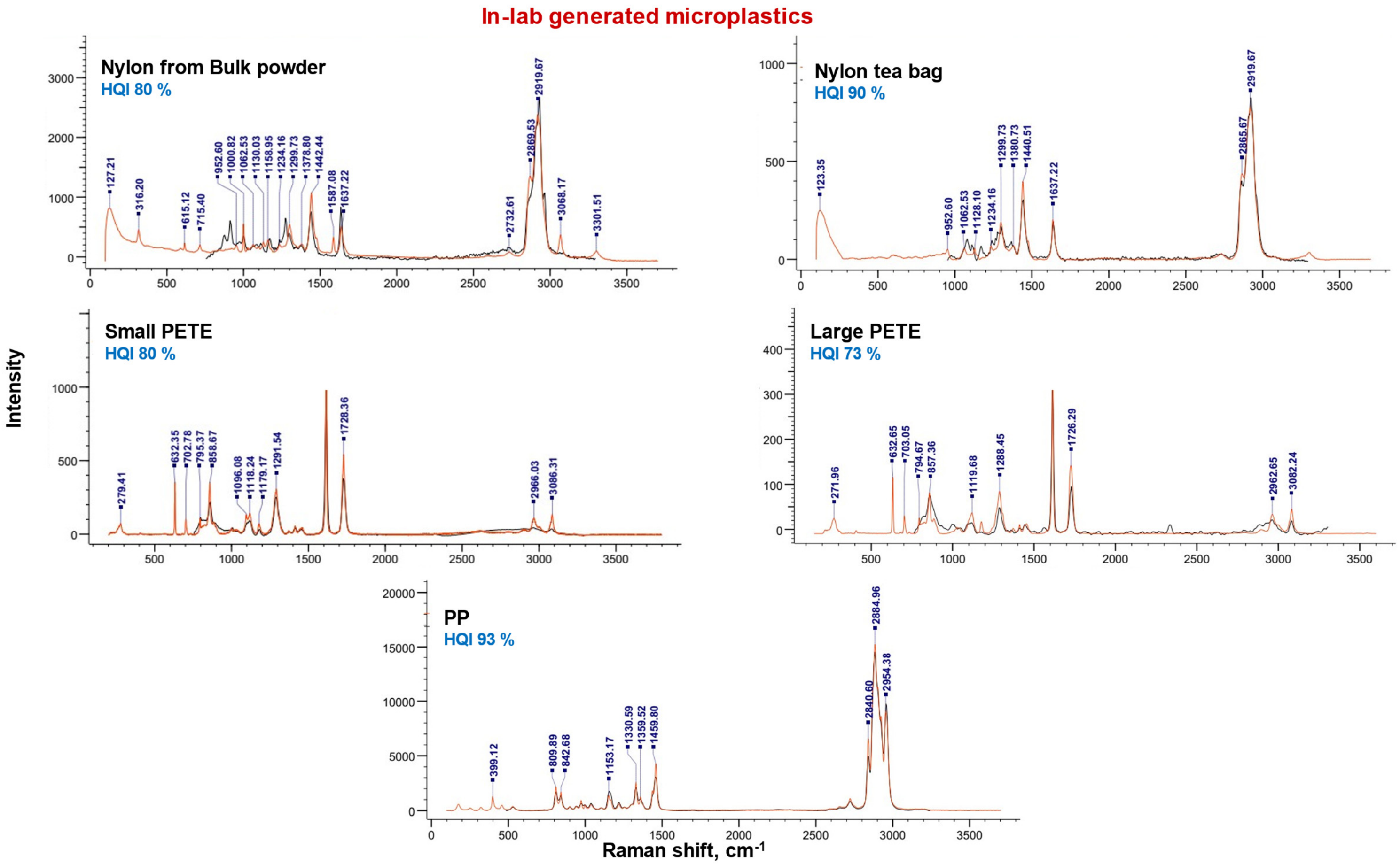

Figure S2. Raman spectra of in-lab generated MPs. The black line represents the spectrum of the sample collected by Labspec6 and the orange line represents the spectrum of the known polymer from the database to which the sample spectrum matched efficiently. The hit quality index (HQI) describes the correlation between the sample spectrum and spectrum of the known polymer.

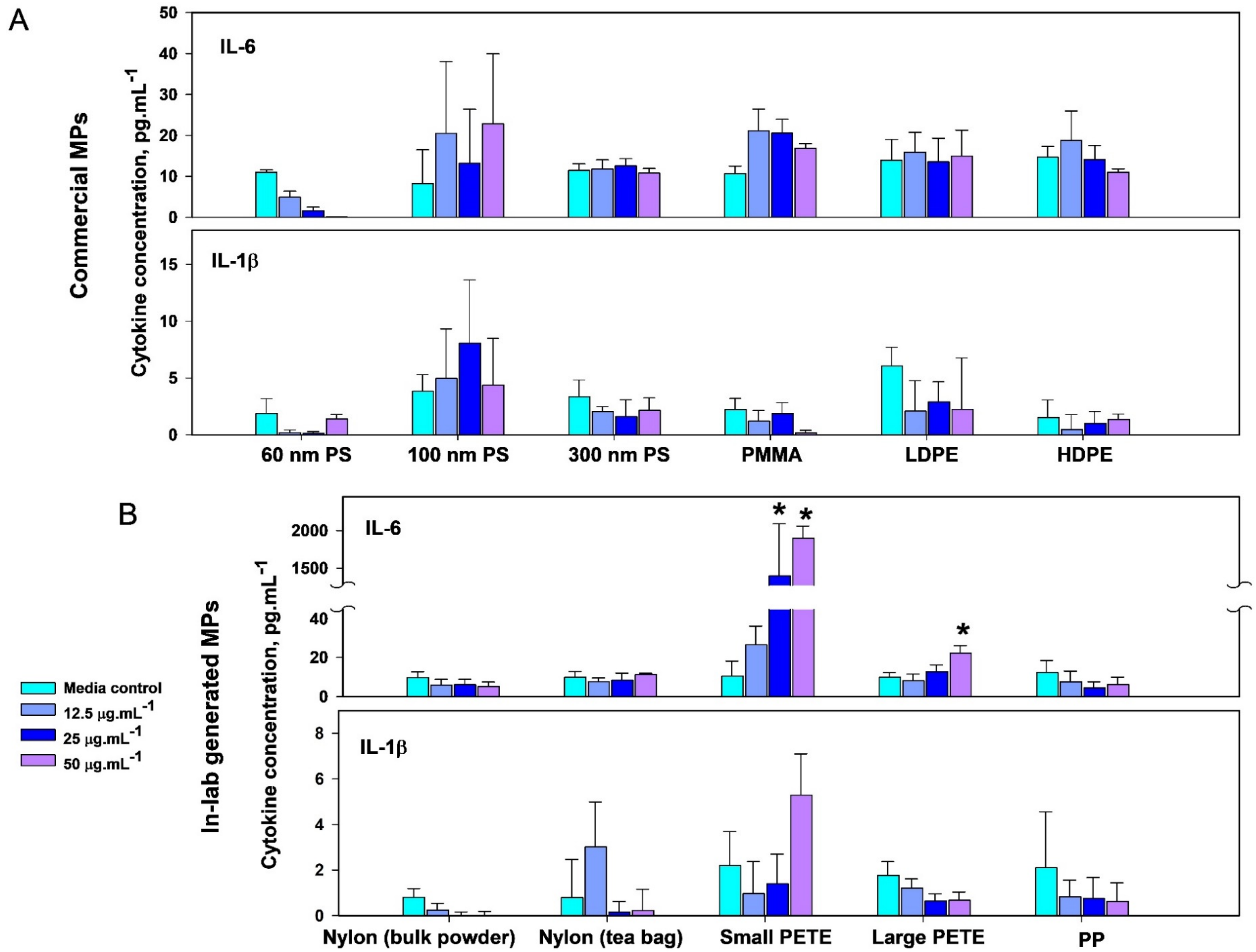

Figure S3. Analysis of pro-inflammatory proteins. IL-6 and IL-1 β expression in cell supernatant post-48 h exposure to 12.5, 25, and 50 μg.mL^{-1} of commercial MPs via single cytokine ELISA (A) and IL-6 and IL-1 β expression levels in supernatant of cells post-48 h exposure to 12.5, 25, and 50 μg.mL−1 of lab-generated MPs via single cytokine ELISA (B). * represents statistical significance. Statistical significance between the exposed samples and matched media control (three biological replicates, two technical replicates) was determined by conducting a two-way ANOVA with a Dunnett's post hoc using $p \leq 0.05$.

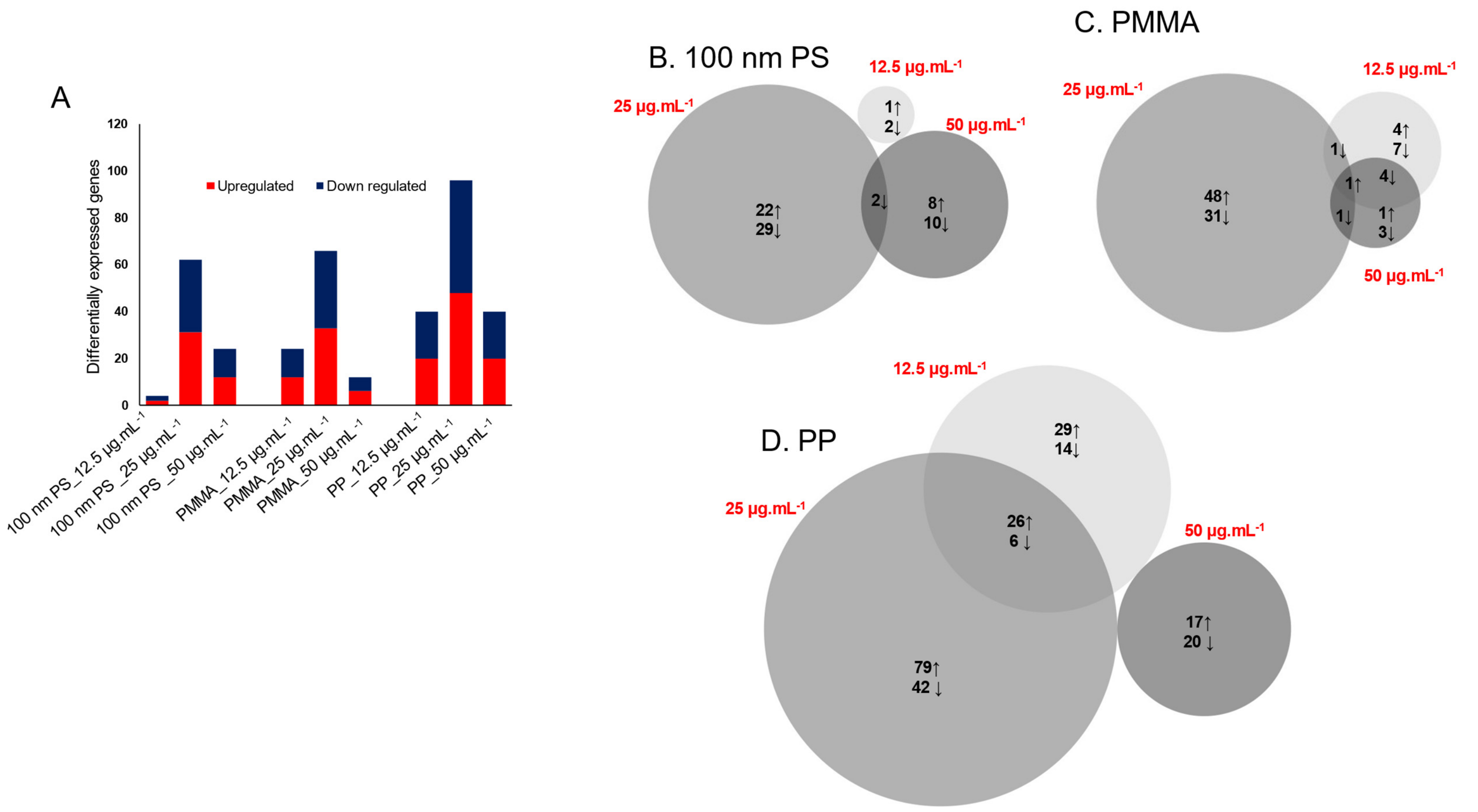

Figure S4. The total numbers of DEGs following 48 h exposure to 100 nm PS, PMMA or PP at 12.5, 25 and 50 µg.mL^{-1} concentrations (A); Venn diagram showing the common DEGs between different dose groups of 100 nm PS (B); Venn diagram showing the common DEGs between different dose groups of PMMA (C) and Venn diagram showing the common DEGs between different dose groups of PP (D). The upward and downward arrows depict up and downregulated genes.

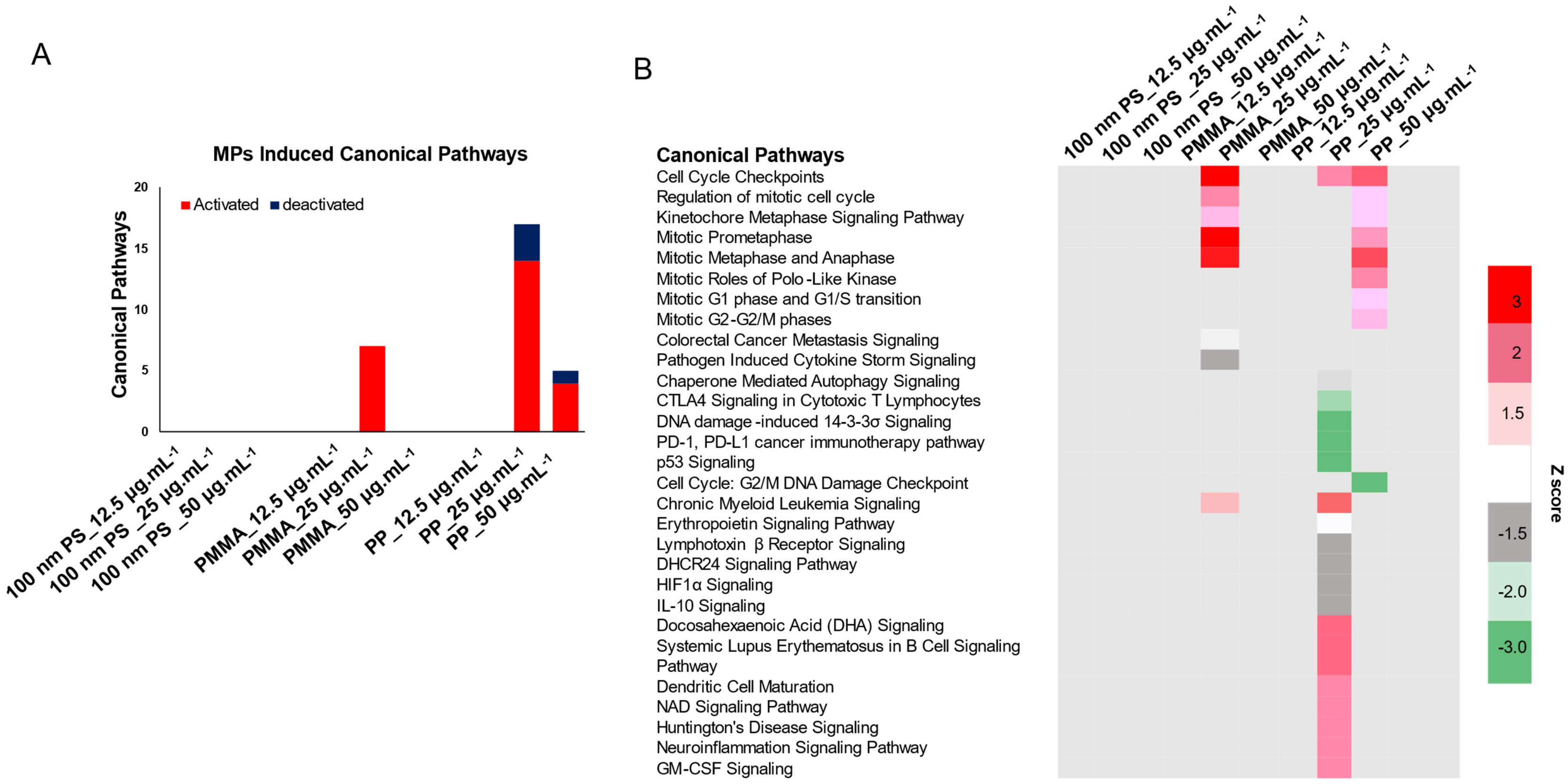

Figure S5. The total number of enriched canonical pathways following 48 h exposure to 12.5, 25 and 50 µg.mL^{-1} doses of 100 nm PS, PMMA and PP (A); The most commonly enriched canonical pathways following 48 h exposure to 100 nm PS, PMMA and PP at 12.5, 25 and 50 µg.mL^{-1} concentrations (B).

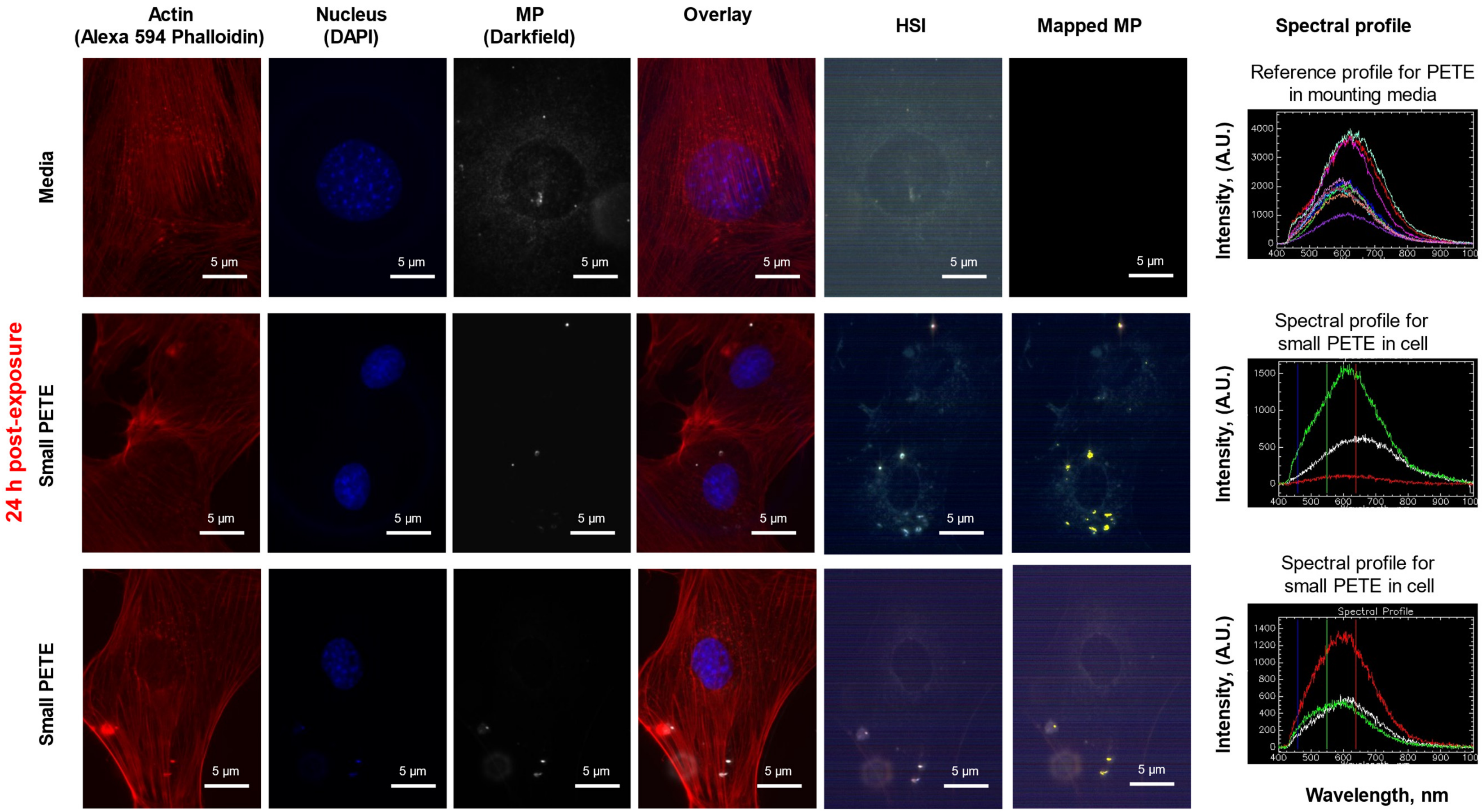

Figure S6. The top and bottom panels show the images of FE1 cells treated with media or 50 µg.mL^{-1} small PETE for 24 h post-exposure, respectively. Vertical panels 1-3 show the distribution of actin (Phalloidin 594 staining, pseudo colored in red), nucleus (DAPI staining, pseudo colored in blue) and particles (EDF images) in the same field of view. The merged images of the epifluorescence with EDF images, the hyperspectral images of the same field of view and the corresponding images of mapped MPs (pseudo colored in yellow) are represented in the vertical panels 4-6 respectively. The hyperspectral images of the PETE particles in mounting media or in cells are shown in the far right panel.

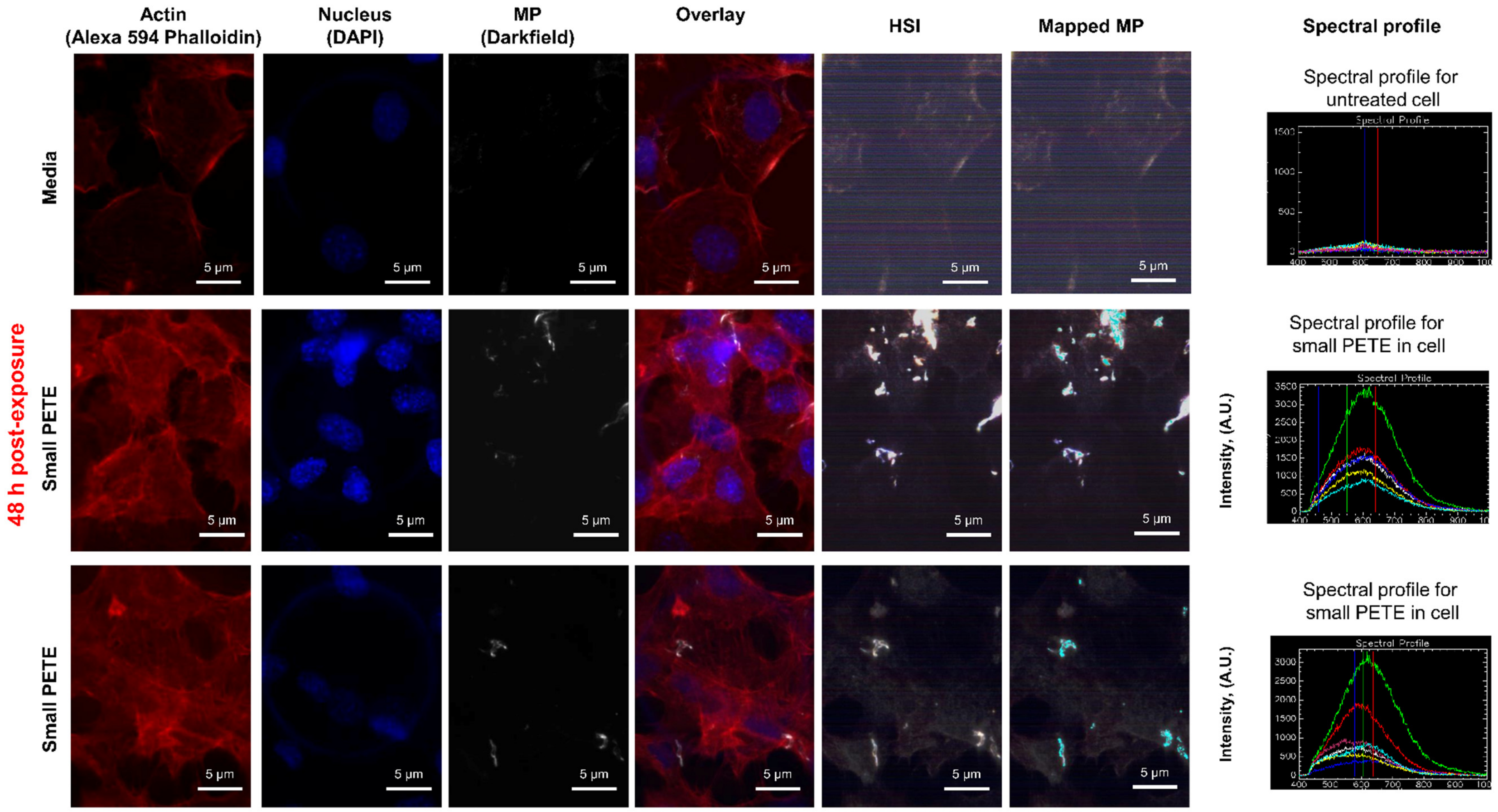

Figure S7. The top and bottom panels show the images of FE1 cells treated with media or 50 µg.mL^{-1} small PETE at 48 h post-exposure. Vertical panels 1-3 show the distribution of actin (Phalloidin 594 staining, pseudo colored in red), nucleus (DAPI staining, pseudo colored in blue) and particles (EDF images) in the same field of view. The merged images of the epifluorescence with EDF images, the hyperspectral images and the corresponding images of mapped MPs (pseudo colored in cyan) are represented in the vertical panels 4-6,respectively. The hyperspectral images of small PETE particles in mounting media or in cells are shown in the far right panel.

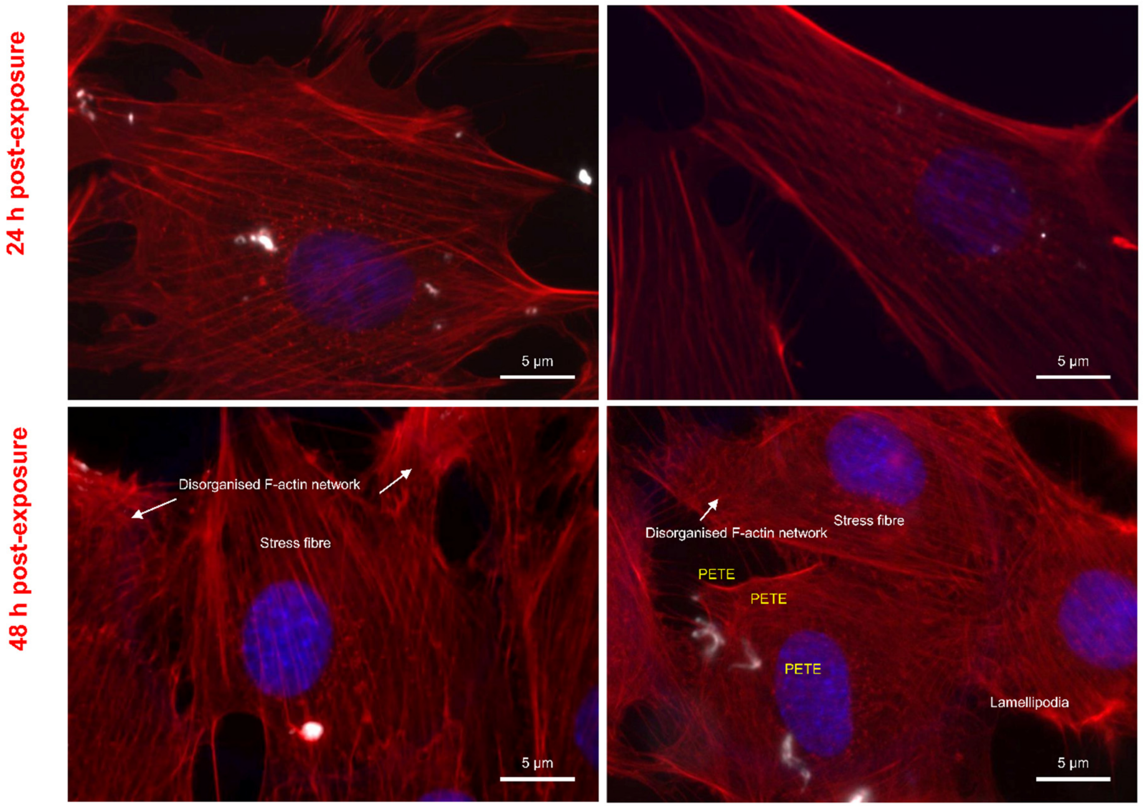

Figure S8. The top and bottom panels show the merged images of the immunofluorescence (Phalloidin 594 staining of actin, pseudo colored in red; DAPI staining of nucleus, pseudo colored in blue) with EDF images of FE1 cells treated with 50 µg.mL^{-1} small PETE at 24h and 48 h post-exposure, respectively.
